# Esophageal luminal stenosis is an independent prognostic factor in esophageal squamous cell carcinoma

**DOI:** 10.18632/oncotarget.14762

**Published:** 2017-01-19

**Authors:** Yu-Shang Yang, Wei-Peng Hu, Peng-Zhi Ni, Wen-Ping Wang, Yong Yuan, Long-Qi Chen

**Affiliations:** ^1^ Department of Thoracic Surgery, West China Hospital of Sichuan University, No. 37, Guoxue Alley, Chengdu, Sichuan, China

**Keywords:** esophageal cancer, outcomes, endoscopy, esophagectomy

## Abstract

**Background:**

Predictive value of preoperative endoscopic characteristic of esophageal tumor has not been fully evaluated. The aim of this study is to investigate the impact of esophageal luminal stenosis on survival for patients with resectable esophageal squamous cell carcinoma (ESCC).

**Methods:**

The clinicopathologic characteristics of 623 ESCC patients who underwent curative resection as the primary treatment between January 2005 and April 2009 were retrospectively reviewed. The esophageal luminal stenosis measured by endoscopy was defined as a uniform measurement preoperatively. The impact of esophageal luminal stenosis on patients’ overall survival (OS) and relation with other clinicopathological features were assessed. A Cox regression model was used to identify prognostic factors.

**Results:**

The results showed that OS significantly decreased in patients with manifest stenotic tumor compared with patients without luminal obstruction (*P*<0.05). Considerable esophageal luminal stenosis was associated with a higher T stage, longer tumor length, and poorer differentiation (all *P*<0.05). In multivariate survival analysis, esophageal luminal stenosis remained as an independent prognostic factor for OS (*P*= 0.036).

**Conclusions:**

Esophageal luminal stenosis could have a significant impact on the OS in patients with resected ESCC and may provide additional prognostic value to the current staging system before any cancer-specific treatment.

## INTRODUCTION

Esophageal cancer is one of the most mortal malignancies worldwide. Most patients die within 1 year of diagnosis, and only 8% to 20% of patients could survive beyond 5 years after the initial diagnosis [1]. Despite improvements in surgical and neoadjuvant therapy, the prognosis still remains poor, with a 5 year survival of approximately 17% and a median survival of 18 months [2]. In the past decades, however, a survival benefit among patients with esophageal cancer receiving neoadjuvant chemoradiotherapy or chemotherapy has been observed [3–5]. An effective and accurate staging system of esophageal cancer is the essential prerequisite to determine the appropriate treatment modalities and predict long-term survival [6, 7]. Upper GI endoscopy is performed routinely in every patient with a suspected esophageal lesion. In the past decade, several studies documented that preoperative endoscopic features, such as tumor length and tumor location, can be utilized as a prognostic factor to predict survival and reflect disease stage. Tumor length measured by endoscopy was repeatedly advocated to be an independent predictor of survival in esophageal cancer [8–11]. Tumor location was chosen to stage the tumor in the recent edition of the AJCC-TNM staging system [12]. However, the prognostic value of endoscopic luminal stenosis measured preoperatively has not been fully evaluated. In the present study, we aimed to access the value of endoscopically measured luminal stenosis in predicting overall survival (OS) in ESCC, and whether it may better select patients with more advanced stages of disease in which neoadjuvant therapy may provide a survival benefit.

## PATIENTS AND METHODS

### Patients

The institutional review board of West China Hospital of Sichuan University approved this study and granted a waiver of the informed consent process. From January 2005 to April 2009, a total of 623 consecutive esophageal cancer patients who underwent esophagectomy at our hospital were reviewed retrospectively. The preoperative staging workup included physical examination, serum biochemistry tests, upper GI endoscopy, chest and upper abdominal CT scan, and abdominal ultrasound. Patients without distant metastasis or definitive evidence of extensive adjacent organ invasion who underwent surgical resection were included in our study. The presence of lymph node enlargement was not a contraindication as long as the nodes were included in the resection. The exclusion criteria included: (1) patients with non-squamous cell carcinoma (*n* = 25); (2) patients receiving neoadjuvant chemoradiation (*n* = 3); (3) patients with incidental finding of M1 stage during operation (*n* = 2); (4) patients with macroscopic or microscopic residual tumor at the surgical margin (*n* = 47); (5) patients who had missing endoscopic measurement data (*n* = 22); (6) patients with surgical mortality, i.e. in-hospital death within 30 days of surgery (*n* = 16).

All the preoperative GI endoscopy were performed with a 9-mm-diameter Olympus gastroscope. Endoscopically measured luminal stenosis was determined after reviewing each patient's preoperative upper GI endoscopy report and classified as two degrees: I, None stenosis or minimal stricture without resistance while passing endoscope; II, significant stenosis allowing endoscopic passage with remarkable resistance or severe stenosis preventing passage of the endoscope through the tumor site.

All patients underwent radical-intent resection. Patients with tumor in middle or lower thoracic esophagus with no evidence of LN involvement in the superior mediastinum or in the neck received esophagectomy *via* left thoracotomy (single incision). Patients with tumor in the middle or upper thoracic esophagus or with possible LN metastasis in the superior mediastinum or neck were operated *via* cervico-thoraco-abdominal (3-field) esophagectomy.

### Pathologic examination

The specimens were preserved in 10% neutral buffered formalin overnight and sent to pathology for examination. Description of the tumor (i.e., appearance, invasion depth, and differentiation) and the lymph nodes were recorded. The surgical and pathologic reports of all the patients were reviewed to ensure accurate staging. Determination of pathologic stages was based on the 7th edition of the American Joint Committee on Cancer TNM staging system.

### Follow-Up

All patients were seen in follow-up at our outpatient department every 3 months in the first 2 years after resection and semiannually thereafter. The follow-up protocol included history taking, physical examination, and chest abdominal CT scans. Upper GI endoscopy, radionuclide bone scans, PET-CT scans, and abdominal ultrasound were arranged if clinically indicated.

The length of survival was defined as the interval in months between the date of operation and the date of death or last follow-up.

### Statistical analysis

Comparisons of categorical data between the two groups were made by chi-square or Fisher's exact probability test. Continuous variables were compared by two-tailed t test. Mann-Whitney U test was applied where required. The OS curves were calculated by the Kaplan-Meier method. Kaplan-Meier survival curves were compared by the log rank test. Univariate and multivariate analyses were performed with the Cox proportional hazards model by SPSS software (version 18.0; SPSS, Chicago, IL). Enter stepwise regression procedure was used. Clinicopathologic factors, such as age, gender, T stage, N stage, tumor length, histologic grading, tumor location, pathological tumor length, and adjuvant therapy, were included in univariate analyses. Variables with P values of < 0.05 in univariate analysis were entered into multivariate analysis.

## RESULTS

**Figure 1:**
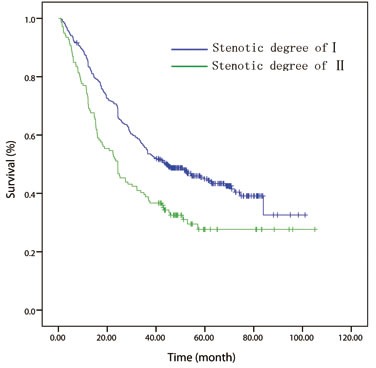
Kaplan-Meier overall survival curves for 508 patients with surgically resected esophageal squamous cell carcinoma stratified by endoscopic luminal stenosis

One hundred and fifteen cases were excluded, as they did not meet the inclusion criteria. Finally, a total of 508 patients were included in the analysis. The mean follow-up time for all 508 patients was 41.2 months (median, 37.5 months; range, 1-105 months). At the last follow-up session, 207 patients were still alive. The 1-, 3- and 5-year OS rates were 85.9%, 50.5%, and 40.2%, respectively. Patients were stratified into groups based on the stenotic degree of esophageal lumen measured by preoperative endoscopy. Accordingly, patients with degree-Istenosis (*n* = 369) had a significantly better 5 year OS rate (45.0% *vs*. 27.7%; *P =* 0.000) than those patients with degree-II stenosis (*n* = 139) (Figure 1). Relation between clinicopathological features and luminal stenosis was listed in Table 1.

**Table 1:** Association between Luminal Stenosis and Clinical Features

Characteristic	Degree-I Stenosis (n=369)	Degree-II Stenosis (n=139)	χ^2^/t/Z value	*P*
Age, year			−0.754*	0.451
Mean	59.84	59.32		
Range	31-80	41-78		
Gender, male/female	306/63	124/15	3.066	0.080
T stage			49.926	0.000
1	74	1		
2	80	15		
3	136	65		
4	79	58		
N stage			4.446	0.217
0	227	73		
1	87	38		
2	45	21		
3	10	7		
Location			0.210	0.899
Upper third	55	23		
Middle third	190	70		
Low third	124	46		
Differentiation			6.712	0.035
Well	23	3		
Moderate	153	48		
Poor	193	88		
Tumor Length, Mean±SD	3.64±2.93	4.55±3.52	−5.166	0.000
Angiolymphatic invasion			0.803	0.370
Yes	327	127		
No	42	12		
Adjuvant			4.257	0.123
Yes	236	99		
No	133	40		

*Z value.

Univariate analyses showed that age (*P =* 0.006), tumor invasion (*P =* 0.000), nodal involvement (*P =* 0.000), tumor length (*P =* 0.001), and luminal stenosis (*P =* 0.000) had a significant effect on OS (Table 2). Tumor invasion (*P =* 0.000), nodal involvement (*P =* 0.000), age (*P =* 0.011), and luminal stenosis (*P =* 0.036) were still independent significant prognostic factors for OS in multivariate analyses (Table 2).

**Table 2:** Univariate and multivariate analyses for overall survival

Characteristic	Univariate analyses			Multivariate analyses		
	HR	95*%CI*	*P*	HR	95*%CI*	*P*
Age	1.020	1.006-1.034	0.006	1.019	1.004-1.034	0.011
Gender	1.398	0.998-1.957	0.051	-	-	-
Tumor Invasion (T_1/2_ *vs*. T_3_ *vs*. T _4_)	1.647	1.422-1.908	0.000	1.438	1.220-1.694	0.000
Nodal Involvement (positive *vs*. negative)	1.972	1.571-2.477	0.000	1.626	1.277-2.070	0.000
Location	1.025	0.870-1.206	0.771	-	-	-
Differentiation	1.159	0.958-1.402	0.128	-	-	-
Tumor Length	1.102	1.040-1.166	0.001	1.017	0.960-1.079	0.562
Adjuvant Therapy	0.755	0.590-0.966	0.025	0.942	0.854-1.040	0.239
Angiolymphatic invasion	1.311	0.926-1.858	0.127	-	-	-
Degree of Stenosis (I *vs*.II)	1.608	1.261-2.051	0.000	1.310	1.017-1.686	0.036

HR, hazard ratio; -, no statistic

**Figure 2:**
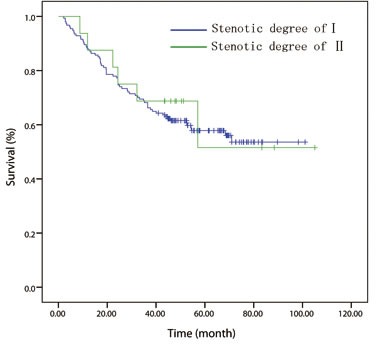
In patient with T1/2 disease, overall survival curve stratified by endoscopically luminal stenosis

**Figure 3:**
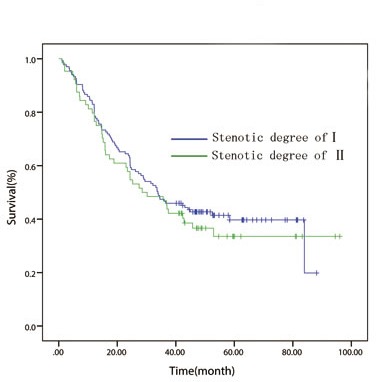
In patient with T3 disease, overall survival curve stratified by endoscopically luminal stenosis

**Figure 4:**
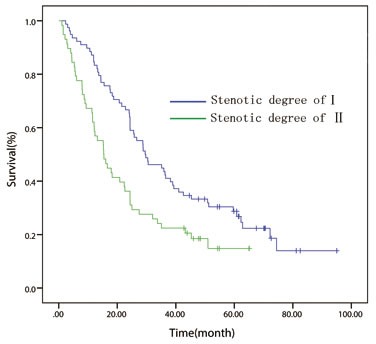
In patient with T4 disease, overall survival curve stratified by endoscopically luminal stenosis

**Figure 5:**
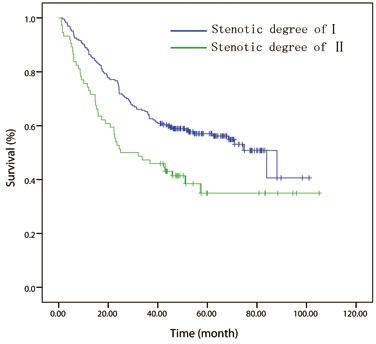
In patients with node-negative disease, overall survival curve stratified by endoscopically luminal stenosis

**Figure 6:**
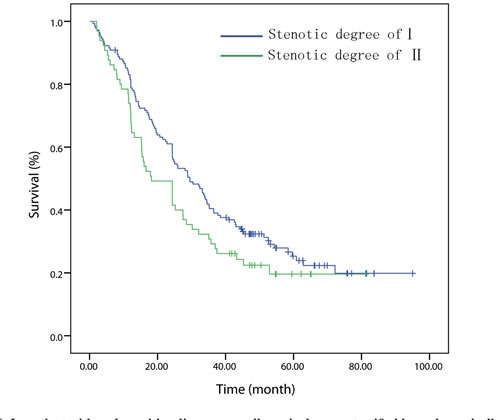
In patient with node-positive disease, overall survival curve stratified by endoscopically luminal stenosis

Patients were further stratified into different groups according to the current TNM staging system in order to assess the effect of esophageal luminal stenosis on the depth of tumor invasion and lymph node status. Patients were divided into T1/2 (*n* = 170), T3 (*n* = 201) groups and T4 (*n* = 137) groups for analysis. In the T1/2 and T3 groups, the difference between the patients with or without luminal stenosis has no statistical significance (*P =* 0.776, Figure 2; *P* = 0.487, Figure 3). In the T4 group, the 5 year OS of degree-I patients was significantly better than patients with degree II (*P = 0.003*, Figure 4). Patients were also divided into node-negative (*n* = 301) and node-positive (*n* = 207) groups for analysis. Trends were observed that degree-I patients had a better OS in ESCC patients regardless of lymph node involvement compared with patients with degree II, but only comparison in the node-negative group achieved statistical significance (node-negative group, *P* = 0.002, Figure 5; positive group, *P* = 0.079, Figure 6).

## COMMENT

Esophageal cancer has been known for its highly malignant nature worldwide. The treatment strategies vary among institutions and change over time. In addition to surgery, neoadjuvant therapy, including chemotherapy and chemoradiotherapy, has been known to provide a survival benefit [3]. However, there is no consensus regarding which group of patients can benefit from neoadjuvant therapy. In order to answer this question, accurate staging is important because treatment modalities are tailored to the stage of the disease.

Besides a tissue biopsy for histological confirmation of malignancy, the preoperative GI endoscopy can provide the macroscopic features of the tumor, such as the location, tumor length, and degree of obstruction. It will assist with treatment plan to carefully record these features [13]. For example, location was suggested as independent staging factors for esophageal cancer and chosen to stage the tumor in the recent edition of the AJCC TNM staging system. Before 1987, esophageal tumor length < 5 cm was categorized as T1 status and > 5 cm as T2 status by the 1983 American Joint Committee on Cancer (AJCC) TNM staging system [14, 15]. Although replaced by depth of esophageal wall invasion in the 1987 version, tumor length was still recognized as a factor correlated with circumferential extent of tumor. However, the prognostic role of luminal stenosis and its predictability of disease stage were rarely emphasized.

Only a few reports have discussed the value of endoscopic luminal stenosis in predicting survival in esophageal cancer. Mariette et al. reported a study of 411 patients to determine the prognostic significance of failure to cross esophageal tumors by endoluminal ultrasound [16]. They observed the median and 5-year survival in patients whose tumors were not crossed was 10 months and 28%, respectively, compared with 24 months and 24%, respectively in patients whose tumors were fully assessed. However, the number of patients in whom EUS failed to cross the primary tumor was small (2.9%) and the consequence was not calculated by multivariable-adjusted models. Poorer prognosis in patient with preoperative tumor stenosis compared with patients without stenosis was reported in three studies by Mariette et al. [17] including 150 patients, Rieu et al. [18] including 120 patients, and Alidina et al. including 97 patients. But the presence of stenosis failed to show as an independent prognostic factor in the multivariate analyses for overall survival in both trials by Rieu et al and Mariette et al. However, in the aforementioned studies, the ratio of tumor stenosis was vary (2.9%-73%) due to the apparent difference in definition of tumor stenosis. The risk of stenosis seems to be relatively high in the ESCC compared with adenocarcinoma [16], while all the previous studies have not grouped the patients by histology. Moreover, the therapeutic approaches of each trial showed considerable heterogeneity. The above results must therefore be interpreted with caution.

The depth of tumor invasion is also a well-established independent prognostic factor for esophageal cancers [6]. In our results, luminal stenosis was associated with more advanced T stage (*P =* 0.000, Table 2). Luminal stenosis remained a prognosticator after controlling for depth of invasion, and its predictive value was significant for T4 lesions in OS.

Lymph node status has been shown to be a strong independent prognostic factor in patients with esophageal cancer [19, 20]. However, luminal stenosis appeared to have a greater impact on lymph node-negative rather than lymph node-positive patients. We also found that a luminal obstruction was associated with higher T stage, larger tumor size, and poorer histological differentiation.

To our knowledge, the present study is the first study to evaluate the value of preoperative measurement of esophageal luminal stenosis in predicting OS in patients with ESCC. Our results validated the prognostic value of endoscopic tumor stenosis and suggested tumor with stenotic degree of II may imply a poor OS in patients with ESCC as compared to degree-I tumor. Several limitations to this study should be mentioned, such as its retrospective nature. The upper GI endoscopy was not performed by a single specialist. Therefore, inter-observer bias may exist. Furthermore, ESCC patients with advanced disease or distant metastasis were not surgical candidates and were not enrolled in the study. Therefore, our results may not be applied comfortably to all patients with ESCC.

In summary, our study suggested that preoperative measurement of endoscopic tumor stenosis can predict OS in ESCC patients who later underwent surgical resection, and the degree of luminal stenosis could be used as an additional effective instrument in identifying those ESCC patients at increased risk of tumor progression. Thus, tumor stenosis should be taken into account when preoperative esophageal tumor staging is performed. Prospective studies may be warranted to further validate the significance of adding clinical tumor obstruction as an additional criterion in the current TNM esophageal staging system.
